# Effects of combined application of fibroblast growth factor (FGF)-2 and carbonate apatite for tissue regeneration in a beagle dog model of one-wall periodontal defect

**DOI:** 10.1016/j.reth.2023.04.002

**Published:** 2023-04-22

**Authors:** Toshie Nagayasu-Tanaka, Jun Anzai, Masahide Takedachi, Masahiro Kitamura, Tatsuhiro Harada, Shinya Murakami

**Affiliations:** a Pharmacology Department, Drug Research Center, Kaken Pharmaceutical Co., LTD., 14, Shinomiya, Minamigawara-cho, Yamashina-ku, Kyoto, 607-8042, Japan; b Department of Periodontology, Osaka University Graduate School of Dentistry, 1-8 Yamadaoka, Suita, Osaka, 565-0871, Japan

**Keywords:** Periodontal regeneration, New bone formation, Micro CT analysis, Fibroblast growth factor-2, Carbonated apatite, Beagle dog one-wall defect

## Abstract

**Introduction:**

There has been an increasing desire for the development of predictive periodontal regenerative therapy for severe periodontitis. In this study, we investigated the effect of the combined use of fibroblast growth factor-2 (FGF-2), a drug for periodontal regeneration approved in Japan, and carbonated apatite (CO_3_Ap), bioresorbable and osteoconductive scaffold, on periodontal regeneration in beagle dog model of one-wall periodontal defect (severe intraosseous defect) for 24 weeks in comparison with CO_3_Ap or vehicle alone.

**Methods:**

One-wall periodontal defects were created (mesiodistal width × depth: 4 × 4 mm) on the mesial portion of the mandibular first molar (M1) of beagle dogs on both side. Mixture of FGF-2 and CO_3_Ap, vehicle and CO_3_Ap, or vehicle alone were administered to the defects and designated as groups FGF-2+CO_3_Ap, CO_3_Ap, and control, respectively. To assess the periodontal regeneration, radiographic analysis over time for 24 weeks, and micro computed tomography (μCT) and histological evaluation at 6 and 24 weeks were performed.

**Results:**

For the regenerated tissue in the defect site, the mineral content of the FGF-2+CO_3_Ap group was higher than that of the CO_3_Ap group in the radiographic analysis at 6–24 weeks. In the context of new bone formation and replacement, the FGF-2+CO_3_Ap group exhibited significantly greater new bone volume and smaller CO_3_Ap volume than the CO_3_Ap group in the μCT analysis at 6 and 24 weeks. Furthermore, the density of the new bone in the FGF-2+CO_3_Ap group at 24 weeks was similar to those in the control and CO_3_Ap groups. Histological evaluation revealed that the length of the new periodontal ligament and cementum in the FGF-2+CO_3_Ap group was greater than that in the CO_3_Ap group at 6 weeks. We also examined the effect of the combined use of the FGF-2 and CO_3_Ap on the existing bone adjacent to the defect and demonstrated that the existing bone height and volume in the FGF-2+CO_3_Ap group remained significantly greater than those in the CO_3_Ap group.

**Conclusion:**

This study demonstrated that the combination of FGF-2 and CO_3_Ap was effective not only in enhancing new bone formation and replacing scaffold but also in maintaining the existing bone adjacent to the defect site in a beagle dog model of one-wall periodontal defect. Additionally, new periodontal tissues induced by FGF-2 and CO_3_Ap may follow a maturation process similar to that formed by spontaneous healing. This suggests that the combined use of FGF-2 and CO_3_Ap would promote periodontal regeneration in severe bony defects of periodontitis patient.

## Highlights

• Combination of FGF-2 with CO_3_Ap enhanced bone formation in dog periodontal defect.
• Accelerating regeneration by FGF-2 is effective since regeneration is limited early.
• Replacement of CO_3_Ap with new bone was accelerated by FGF-2.
• Density of bone formed by combination was equivalent to that of spontaneous healing.
• Combination was effective in maintaining the existing bone adjacent to defect.

## 1 Introduction

For severe periodontitis, in which serious alveolar bone resorption occurs, periodontal surgery is performed to exfoliate the gingiva and remove the lesions infected with periodontopathic bacteria, including the alveolar bone. However, although surgery is successfully performed, functional regeneration of lost periodontal tissue cannot be expected as the removed bone space is rapidly occupied by the gingival tissue to interfere with the neogenesis of the alveolar bone, periodontal ligament (PDL), and cementum.

Generally, the ultimate goal of periodontal therapy is functional regeneration of lost periodontal tissue. To this aim, some periodontal regenerative procedures with bone substitute, guided tissue regeneration (GTR) membrane and enamel matrix derivative, have been developed to secure regenerative spaces and/or activate periodontal regeneration [1]. However, the success of these therapies depends on the skill of the surgeon and severity of tissue damage. Therefore, unmet medical needs for effective and functionally regenerative therapies for severe periodontitis remain high.

Fibroblast growth factor-2 (FGF-2) is a well-known growth factor that facilitates angiogenesis [2]. Furthermore, it strongly induces proliferation and migration of mesenchymal cells responsible for the regeneration of the alveolar bone, PDL, and cementum [[3], [4], [5], [6], [7], [8], [9]]. We demonstrated that FGF-2 stimulates neogenesis of the alveolar bone, PDL, and cementum in experimentally prepared beagle dog [[10], [11], [12], [13]] and non-human primate [14] models of periodontal defect. In addition, we conducted clinical trials of intrabony defects in patients with periodontitis and demonstrated that FGF-2 was superior to the vehicle alone in terms of the percentage of bone fill in modified Widman periodontal surgery [[15], [16], [17]]. Based on these data, FGF-2 hydroxypropyl cellulose (HPC) formulation was approved in Japan and is now commercially available as a drug for periodontal regeneration (Regroth® Dental Kits), with emerging reports of its efficacy. For example, the 6-year postoperative radiograph of a patient with aggressive periodontitis who was treated with FGF-2 revealed significant development of the alveolar bone in comparison with the first visit [18]. Furthermore, the class 2 furcation defect was completely filled with regenerated bone 6 months after the FGF-2 administration [19]. However, severe defects, such as one-wall periodontal defect, or class 3 furcation involvement may require the combined use of FGF-2 and scaffold as the drug formulation itself does not have the ability to maintain the space for periodontal regeneration.

To treat such severe bony defects or horizontal bone resorption, osteoconductive scaffold has been applied to the defects to maintain the regeneration space. Hydroxyapatite (HAp), β-tricalcium phosphate (β-TCP), and carbonated apatite (CO_3_Ap) are typical artificial scaffolds clinically used as bone substitutes. Whereas β-TCP spontaneously dissolves under physiological condition without osteoclastic resorption [20], CO_3_Ap is an inorganic component of the bone and, unlike β-TCP, dissolves only in a weakly acidic environment where osteoclasts absorb bone in response to bone remodeling [21]. Furthermore, CO_3_Ap has been reported to promote osteoblastic differentiation of human bone marrow cells earlier than HAp owing to its high osteoinductivity [22]. Because of these properties, CO_3_Ap has been reported to maintain its shape even in the body fluid and to hold a space for regeneration under the conditions of post-surgery defect sites [21,23]. Thus, it is considered to be an artificial scaffold with numerous advantages and the first product (Cytrans®) in Japan to be approved for use in dental implant and periodontal surgeries. However, its efficacy when used in combination with FGF-2 in severe periodontitis models is not fully understood.

Focusing on the characteristics of CO_3_Ap, Kitamura et al. conducted a clinical study of the combined use of FGF-2 and CO_3_Ap in 10 periodontitis patients with intrabony defect (mean bone defect depth: 5.7 mm) to evaluate the safety and efficacy of such a combination [24]. As a result, no adverse events associated with the combined use of FGF-2 and CO_3_Ap occurred 36 weeks after administration. Furthermore, regeneration of severe alveolar bone defects, where sufficient periodontal regeneration is unlikely with Regroth® monotherapy, was observed. However, the change/maturation of the intra-defect tissue and volume of the new bone could not be investigated due to the use of radiographies for the evaluation.

In this study, the effects of the combined use of FGF-2 and CO_3_Ap on periodontal regeneration were investigated using a beagle dog model of one-wall periodontal defect. In particular, to analyze the interaction between CO_3_Ap and FGF-2 in the new bone formation, the bone and scaffold in the defect site were evaluated over time for 24 weeks in a radiographic analysis. Furthermore, the quantity and quality of the bone and scaffold were separately evaluated *via* μCT at 6 and 24 weeks. Histological evaluation was also performed to assess the condition of the tissue.

## 2 Methods

### 2.1 Preparation of test substances

For FGF-2, Regroth® Dental Kit was used, which is a 3% HPC solution containing 0.3% human recombinant FGF-2 (Kaken Pharmaceutical Co. Ltd., Tokyo, Japan). For the vehicle control, a 3% HPC solution itself was used. HPC was added to facilitate the administration owing to its high viscosity. CO₃Ap (Cytrans® granules; M size; GC Co. Ltd., Tokyo, Japan) was provided by GC Co., Ltd., under a joint research project.

### 2.2 Animals

A total of 20 female TOYO beagle dogs (27–42 months old, weighing 8.2–10.8 kg) were used. They were housed individually and allowed to move freely in stainless-steel cages under the following conditions: 18°C-26°C temperature, 30%–70% humidity, and 12-h lighting (07:00–19:00). The animal room and cages were cleaned daily. The dogs were taken out of their cages and allowed to exercise freely in the animal room twice a day to reduce their stress. Furthermore, they were provided with 250 g of solid food per day and filtered tap water *ad libitum*. After the tooth extraction and surgery, the animals were given about 800 g of soft food per day for 1 week. No animals became ill or died prior to the experimental endpoint. This study was approved by the Animal Research Ethics Committee of Kaken Pharmaceuticals Co., Ltd. (Permit Number: K19-140, Date of approval: Aug 28, 2019).

The in-house regulations at Kaken comply with Japan's Act on Welfare and Management of Animals, and the related international and domestic guidelines are followed. The ethics committee established based on the in-house regulations reviews whether all animal experimental protocols are written based on the “3Rs (Replacement, Reduction, and Refinement) principles” before an experiment may begin and implements self-inspections and assessments of the animal experiment processes and the operations facility. In addition, the laboratory animal facilities of Kaken have been certified by the Japan Pharmaceutical Information Center (Tokyo, Japan), which assesses and verifies compliance with the “Basic policies for the conduct of animal experimentation in the Ministry of Health, Labour and Welfare (Japan)”.

### 2.3 Periodontal surgery and administration of test substances

All beagle dogs were subjected to an artificially created one-wall periodontal defect. After the dogs were anesthetized subcutaneously with xylazine (20 mg/dog), intravenously with pentobarbital (10 mg/kg), and intragingivally with 2% lidocaine and 0.00125% adrenaline, the right and left fourth premolars (P4) in the mandible were extracted. For pain relief, meloxicam (0.2 mg/kg) was administered subcutaneously. Six months later, the beagle dogs were anesthetized as described above, a mucoperiosteal flap was raised after the incisions were made closer to the lingual side of the alveolar crest. One-wall periodontal defects (mesiodistal width × depth: 4 × 4 mm) were surgically created on the mesial portion of the mandibular first molar (M1) on both sides (Supplemental Figs. S1a–d). The supporting bone and cementum of the teeth were removed using steel burs, and the exposed root surfaces of the teeth were smoothened using Gracey curettes. The group composition and number of defects are depicted in Supplemental Fig. S1e. A control group was established to establish the degree of regeneration in this model. In the control group, the vehicle (150 μL/site) was administered to both defects in four dogs. In the CO_3_Ap group, after mixing CO_3_Ap and the vehicle (0.25 g: 200 μL), the mixture was administered to defects. In the FGF-2+CO_3_Ap group, after mixing CO_3_Ap and FGF-2 in the same ratio, the mixture was administered to defects. Each group was placed on either side of the same individual to lower the effect of individual differences in 16 dogs. The gingiva was partially sutured before test substance administration to avoid leakage during administration. Multiple single sutures were used, and the incision surfaces were in close contact. Penicillin (40,000 U/dog) and streptomycin (200 mg/dog) were administered subcutaneously to prevent infection following surgery. The treated sites were observed every day until the wounds healed. Dental calculus was removed at the time of taking the X-ray photographs as oral care following surgery.

### 2.4 Radiographic analysis

Under general anesthesia, radiographic images of the defect site and aluminum step wedge (1–7 mm thick) were obtained in the buccolingual direction using dental X-ray film on an X-ray apparatus (CMBW-2; Softex Co., Ltd., Tokyo, Japan) immediately after administration (0 week) and 1 week, and 3 weeks, and every 3 weeks after 3 weeks of administration (Supplemental Fig. S1f and Supplemental Fig. S2a). Image-Pro Plus (Media Cybernetics Inc. Rockville, Md, USA) was used to digitize the radiographic images, and then the mineral area (Supplemental Fig. S2a green square) was enclosed in the granules and/or new bone above the defect (Supplemental Fig. S2a green solid line), the root surface and the existing bone around the defect (Supplemental Fig. S2a green dotted line). The mineral density in the mineral area was converted to the thickness of an aluminum step wedge as a density standard. In addition, the height of the existing bone on the mesial side of the defect (Supplemental Fig. S2a yellow solid line) were evaluated using the aforementioned software. The mineral content was calculated by multiplying the mineral density and mineral area of the defect.

### 2.5 Preparation of tissue specimens and μCT analysis

Under general anesthesia, 10 beagle dogs each were euthanized by exsanguination at 6 and 24 weeks after administration, and the tissues containing the defect site were harvested and fixed in neutral-buffered 10% formalin (control group: n = 4 from two dogs, CO_3_Ap and FGF-2+CO_3_Ap groups: each n = 8 from eight dogs).

The μCT images of the defect site (mesiodistal width: 4 mm) and the existing bone on the mesial side of the defect (mesiodistal width: 1 mm) were obtained *via* microfocus X-ray CT (ScanXmate-E090S40 *in vivo*; ComscanTecno Co., Ltd., Yokohama, Japan) and analyzed using TRI/3D-BON (Ratoc System Engineering, Tokyo, Japan) (Supplemental Fig. S2 b). In the defect site, the volume of the new bone and CO_3_Ap, separated from the new bone based on the difference in X-ray transparency, and the mineral density of the new bone were evaluated. In the existing bone region, the bone volume (BV), tissue volume (TV: volume of bone plus bone marrow cavity), and BV/TV were calculated.

### 2.6 Histological analysis

After decalcification, the tissues were paraffin-embedded using common methods. Serial sections were cut in the mesiodistal plane and stained with Azan. Histometric measurements were performed using the VS120 and VS-ASW analysis software (ver. 2.9, Olympus Co., Tokyo, Japan) to evaluate the area of the new bone including CO_3_Ap in the defect site, the height of the existing bone on the mesial side of the defect, and the length of the new PDL and cementum from the defect bottom.

### 2.7 Statistical analysis

The mean and standard deviation (SD) were calculated for each measurement. To evaluate the effect of the combined use of FGF-2 and CO_3_Ap, the differences between the CO_3_Ap and FGF-2+CO_3_Ap groups at each time point were analyzed using paired *t*-test. The length of the new PDL and cementum were assessed by a Wilcoxon's rank sum test because of their non-normal distribution in this study. The significance level was set to 5%. All statistical analyses were conducted using JMP (ver. 15.0, SAS Institute Inc., NC, USA).

## 3 Results

### 3.1 Time course of mineral density, area, and content in the defect site and the height of the existing bone on the mesial side of the defect in the radiographic analysis

**Fig. 1:**
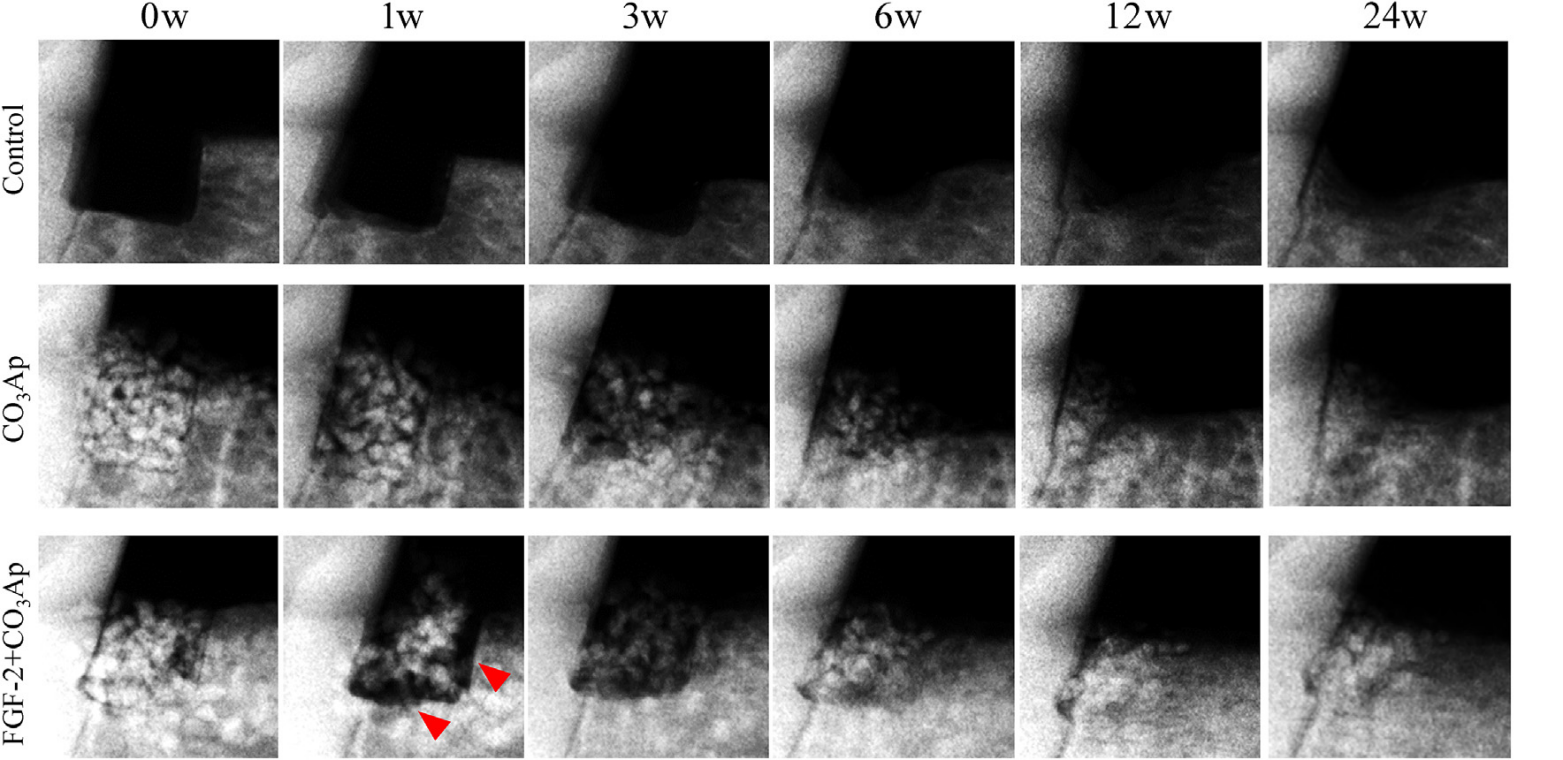
**Representative X-ray images**. Changes in X-ray images over time in the control, CO_3_Ap, and FGF-2+CO_3_Ap groups. Representative X-ray images taken at 0, 1, 3, 6, 12, and 24 weeks (w) after administration are presented. Some images are flipped horizontally. Red arrowheads: gaps without granules were detected along the mesial aspect and the bottom of the defects.

**Fig. 2:**
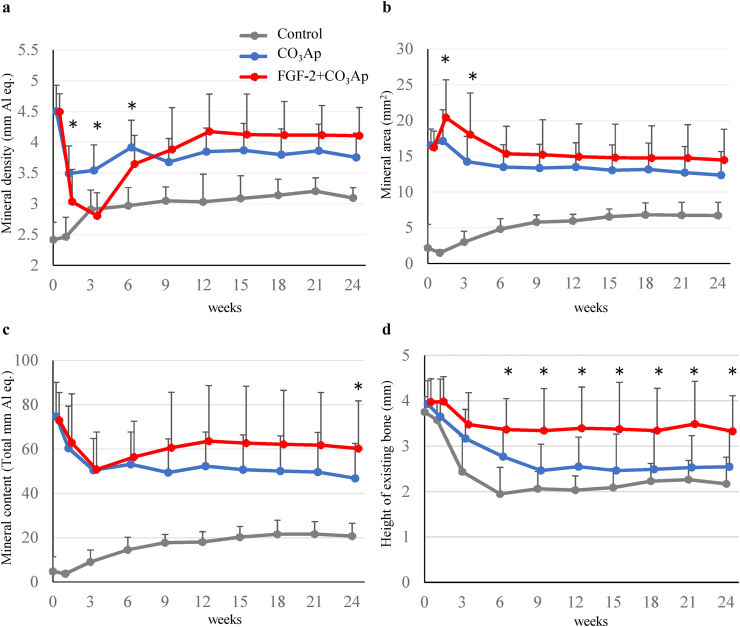
**Time course of the mineral density, area, and content in the defect site and the height of the existing bone on the mesial side of the defect**. The mineral density (a), mineral area (b), and mineral content (c) of the defect site and the height of the existing bone on the mesial side of the defect (d) were evaluated. Control, gray line; CO_3_Ap, blue line; FGF-2+CO_3_Ap, red line. All results are expressed as mean +SD, Control, n = 8; CO_3_Ap, n = 16; FGF-2+CO_3_Ap, n = 16 (0–6 weeks). Control, n = 4; CO_3_Ap, n = 8; FGF-2+CO_3_Ap, n = 8 (9–24 weeks). ∗: *P* < 0.05 (CO_3_Ap vs. FGF-2+CO_3_Ap, paired *t*-test at each time).

In the control group, formation of a new bone at the defect site was observed along the mesial existing bone and the root surface; however, the amount of bone formation was limited (Fig. 1). The mineral density, area, and content of the defect site in the control group slightly increased, although these remained low (Fig. 2a–c).

In the CO_3_Ap and FGF-2+CO_3_Ap groups, CO_3_Ap granules disappeared over time, and the X-ray opacity of the spaces between each of the CO_3_Ap granules increased (Fig. 1). Especially, in the FGF-2+CO_3_Ap group, a gap without granules was observed along the mesial aspect and the bottom of the defects at 1 week (Fig. 1 red arrowheads), and the X-ray opacity of the gap increased at 3–12 weeks. The quantitative results presented in Fig. 2 indicated that the mineral density in the CO_3_Ap group decreased at 1 week and remained similar thereafter, whereas that in the FGF-2+CO_3_Ap group exhibited a greater decrease at 3 weeks, followed by a change toward increase, with higher density than the CO_3_Ap group at 9 weeks onwards (Fig. 2a). The mineral area in the CO_3_Ap and FGF-2+CO_3_Ap groups increased at 0–1 week, then gradually decreased at 6 weeks, and remained similar thereafter (Fig. 2b). The increase was significantly greater in the FGF-2+CO_3_Ap than in the CO_3_Ap group at 1 and 3 weeks. The mineral content, calculated by mineral density and area, in the FGF-2+CO_3_Ap group was higher than those in the CO_3_Ap group at 9 weeks onwards (Fig. 2c).

The height of the existing bone on the mesial side of the defect decreased in all groups (Fig. 2d). However, the decrease in the FGF-2+CO_3_Ap group was the smallest, and the height of the existing bone in the FGF-2+CO_3_Ap group remained significantly greater than that in the CO_3_Ap group at 6 weeks onwards.

### 3.2 Total mineral volume, volume of CO_3_Ap, and the new bone in the defect site

**Fig. 3:**
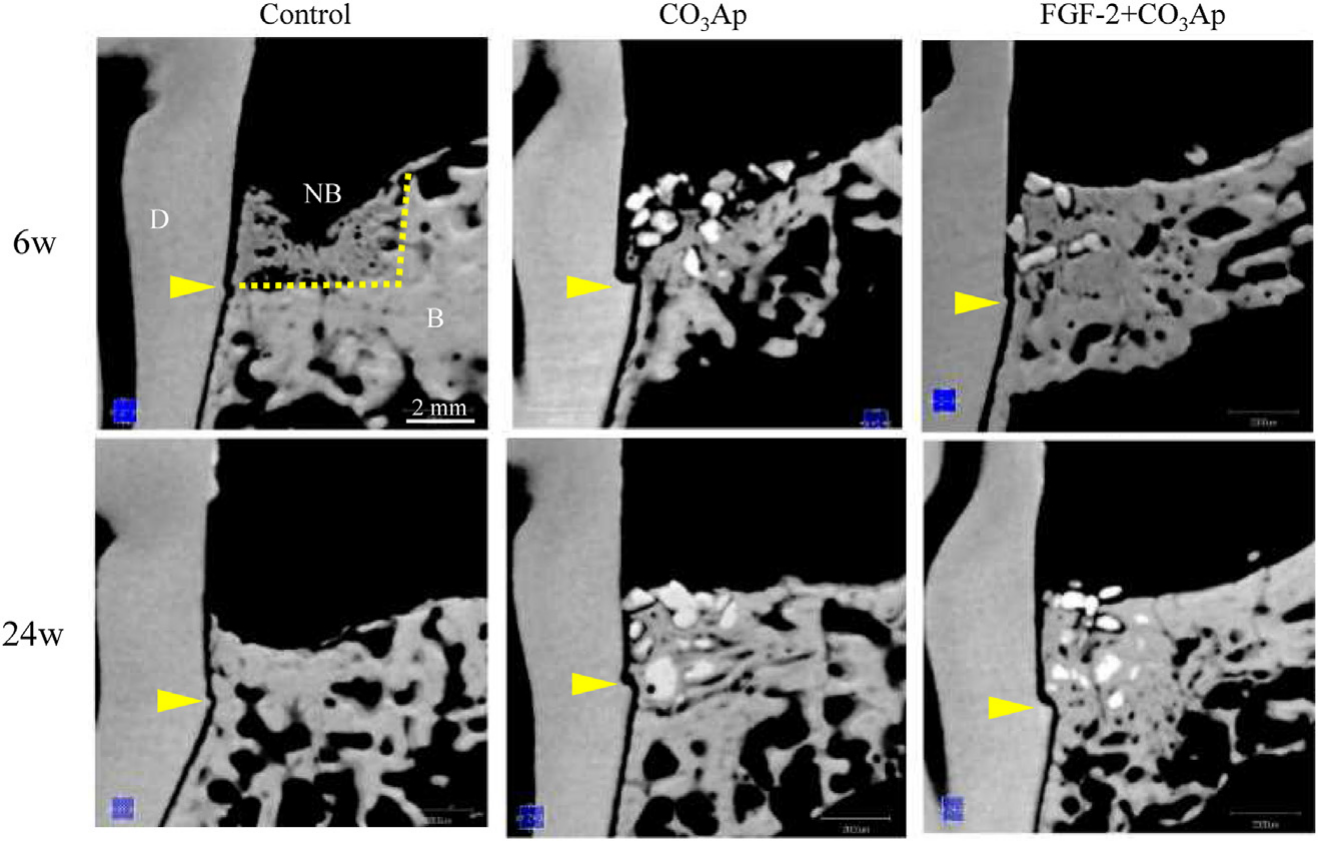
**Representative μCT images**. Sectional images of the central part of the defect site and surrounding tissue were viewed from the buccal side. Upper column, 6 weeks; lower column, 24 weeks. D: dentin, NB: new bone, B: existing bone, yellow arrowheads: defect bottom, yellow dot line: the border between the defect site and the existing bone. Scale bar represents 2 mm.

Sectional images of the central part of the defect site at 6 and 24 weeks obtained *via* μCT are presented in Fig. 3. In the control group, the new bone formation was insufficient to fill the defect, and a decrease in the height of the existing bone was observed at 24 weeks. In the CO_3_Ap group, the new bone formed around the granule, and free granules were present on the coronal side. Furthermore, a decrease in the height of the existing bone was observed at 6 weeks. The formation of the new bone on the coronal side progressed at 24 weeks. In the FGF-2+CO_3_Ap group, a new bone formed surrounding the granules up to the coronal side, and the height of the new bone was the same as that of the existing bone at 6 weeks, which remained the same at 24 weeks.

**Fig. 4:**
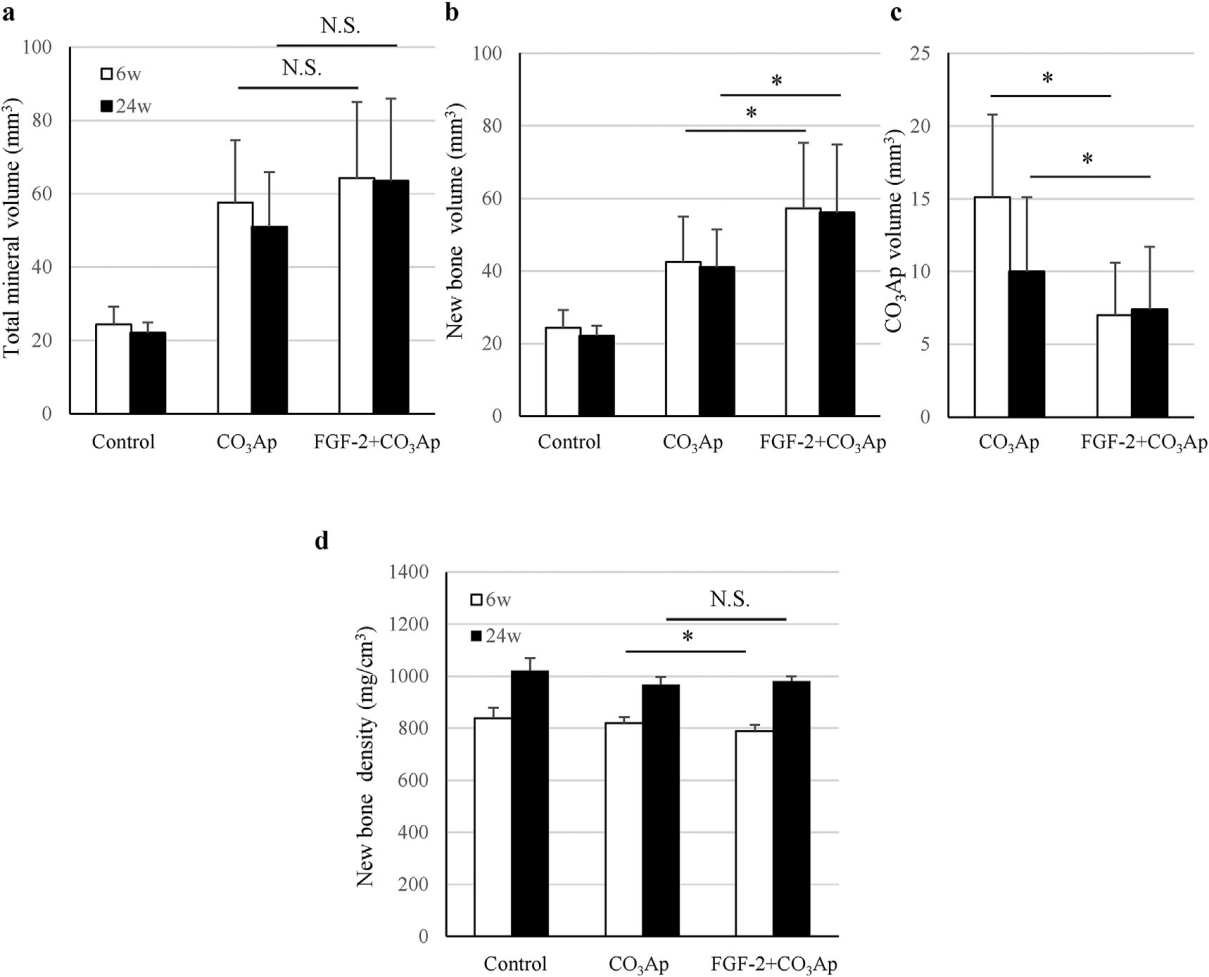
**Mineral and new bone volume and new bone density in the defect site**. The total mineral volume (a), new bone volume (b), CO_3_Ap volume (c), and new bone density (d) were measured in the μCT images. All results are expressed as mean +SD. Control, n = 4; CO_3_Ap, n = 8; FGF-2+CO_3_Ap, n = 8 at each time (white column: 6 weeks, black column: 24 weeks). ∗: *P* < 0.05, NS: not significant (CO_3_Ap vs. FGF-2+CO_3_Ap, paired *t*-test at each time).

Quantitative analysis revealed that the total mineral volume and new bone volume in the CO_3_Ap and FGF-2+CO_3_Ap groups were high than those of the control groups at 6 and 24 weeks (Fig. 4a and b). In addition, the new bone volume of FGF-2+CO_3_Ap group was significantly higher than that of the CO_3_Ap group (Fig. 4b), whereas the CO_3_Ap volume of the FGF-2+CO_3_Ap group was significantly lower than that of the CO_3_Ap group at each time point (Fig. 4c). When the time points were compared, the volume of the new bone at 6 weeks was similar to that at 24 weeks in all groups.

### 3.3 Density of the new bone in the defect site

The density of the new bone in the FGF-2+CO_3_Ap group at 6 weeks was significantly lower than that in the CO_3_Ap group (Fig. 4d). However, the density of the new bone in the FGF-2+CO_3_Ap group at 24 weeks was similar to that in the control and CO_3_Ap groups, and no significant difference was observed between the CO_3_Ap and FGF-2+CO_3_Ap groups. When the time points were compared, the density of the new bone in all groups at 24 weeks was higher than that at 6 weeks.

### 3.4 BV, TV, and BV/TV in the existing bone region

The effect of the combined use of the FGF-2 and CO_3_Ap on the existing bone adjacent to the defect was examined.

**Fig. 5:**
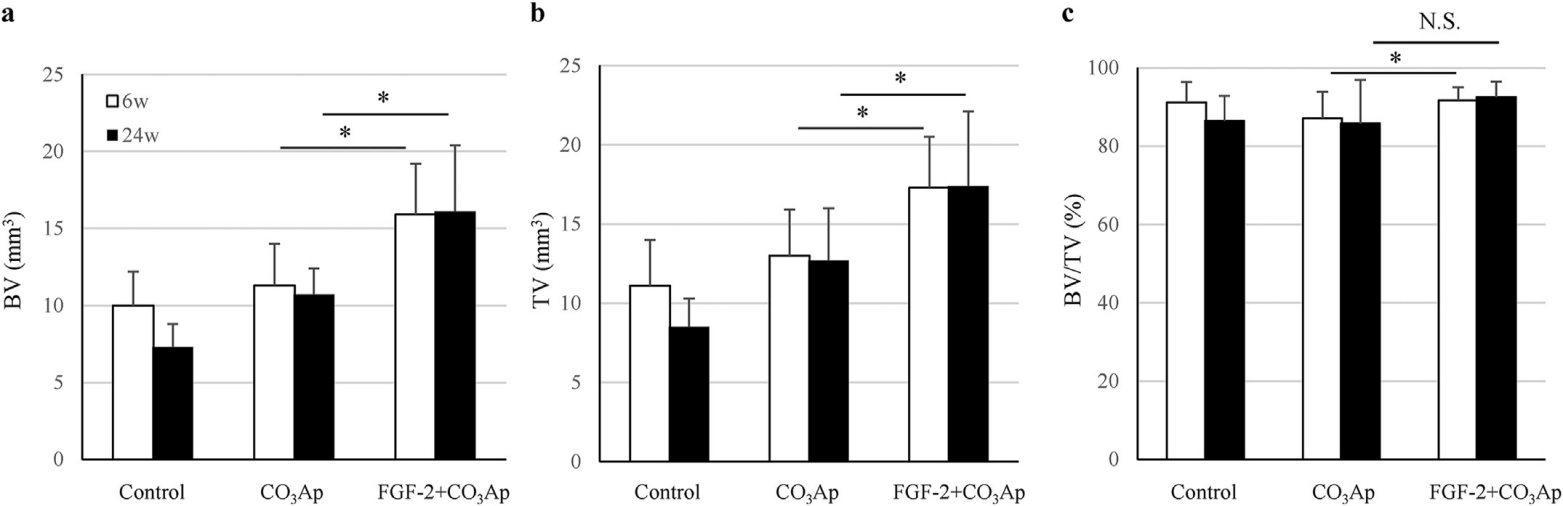
**BV, TV, and BV/TV in the existing bone region**. In the existing bone region, the BV (a), TV (b), and BV/TV (c) were measured in the μCT images. All results are expressed as mean +SD, Control, n = 4; CO_3_Ap, n = 8; FGF-2+CO_3_Ap, n = 8 at each time (white column: 6 weeks, black column: 24 weeks). ∗: *P* < 0.05, NS: not significant (CO_3_Ap vs. FGF-2+CO_3_Ap, paired *t*-test at each time).

The BV and TV in the existing bone region located on the mesial side of the defect site were the highest in the FGF-2+CO_3_Ap group and the lowest in the control group. Furthermore, the BV and TV in the FGF-2+CO_3_Ap group were significantly higher than those in the CO_3_Ap group at 6 and 24 weeks (Fig. 5a and b).

Although the BV/TV in the FGF-2+CO_3_Ap and CO_3_Ap groups was statistically different at 6 weeks, no difference was observed in all groups at 24 weeks (Fig. 5c). This result suggests that the existing bone quality in all groups had similar properties with the passage of time.

### 3.5 Histological findings and quantitative analysis in the defect site

**Fig. 6:**
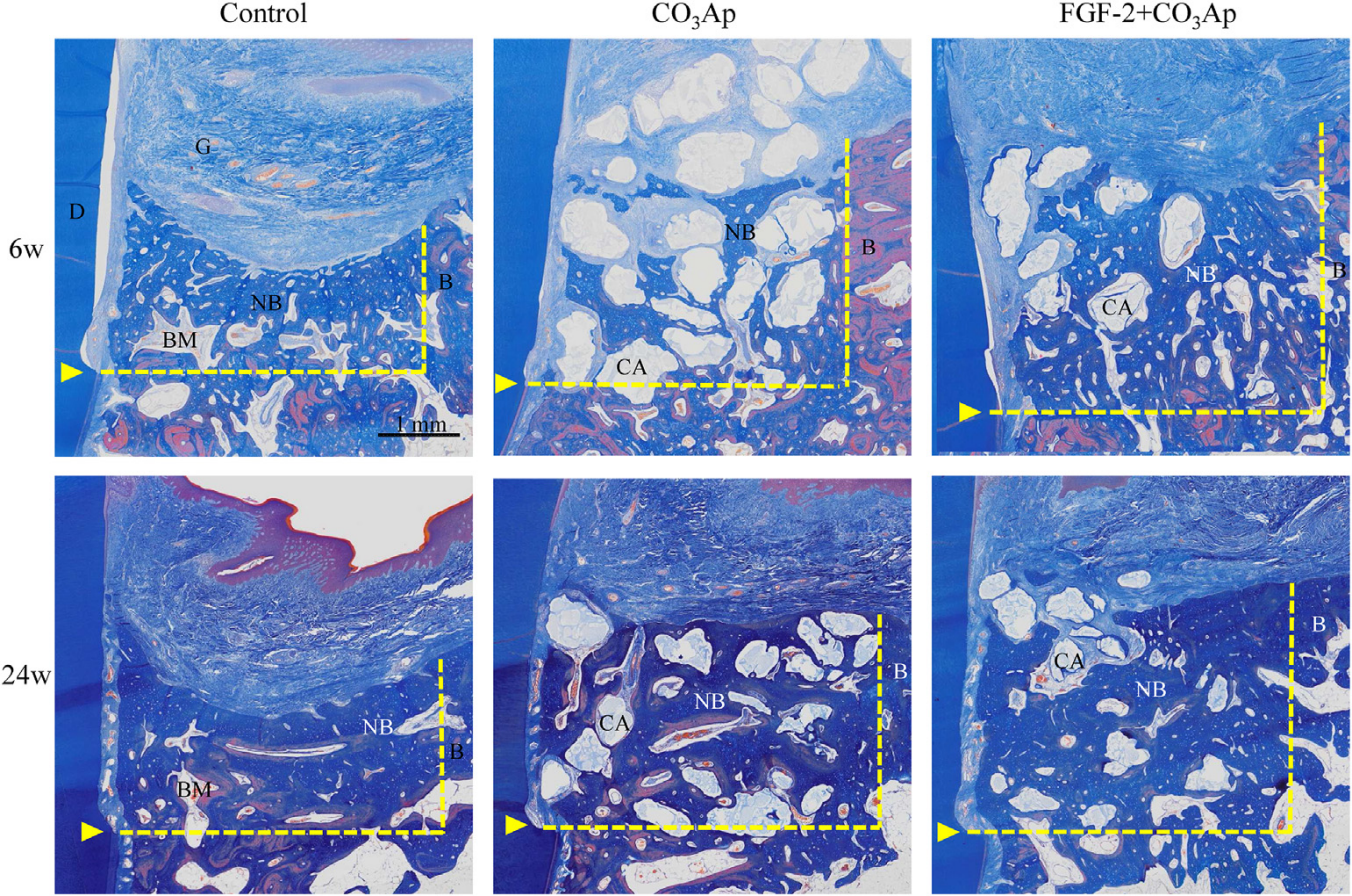
**Histological overview of the regenerated tissue in the defect site**. Representative Azan staining images of the central part of the defect site and surrounding tissue are presented. Upper column: 6 weeks, lower column: 24 weeks. D: dentin, G: gingival connective tissue, NB: new bone, BM: bone marrow, B: existing bone, CA: CO_3_Ap, yellow arrowheads: defect bottom, yellow dot line: the border between the defect site and the existing bone. Scale bar represents 1 mm.

Histological images of the entire bone defect at 6 and 24 weeks are presented in Fig. 6. In all groups, a new bone in the defect site and a new connective tissue on the root surface were found. However, in the control group, the new bone had a concave shape at both 6 and 24 weeks, and the height of the existing bone decreased. In the CO_3_Ap group, a new bone with granules occupied the defect. Furthermore, the defect of the FGF-2+CO_3_Ap group had less CO_3_Ap granules and was filled with more new bones than the CO_3_Ap group.

**Fig. 7:**
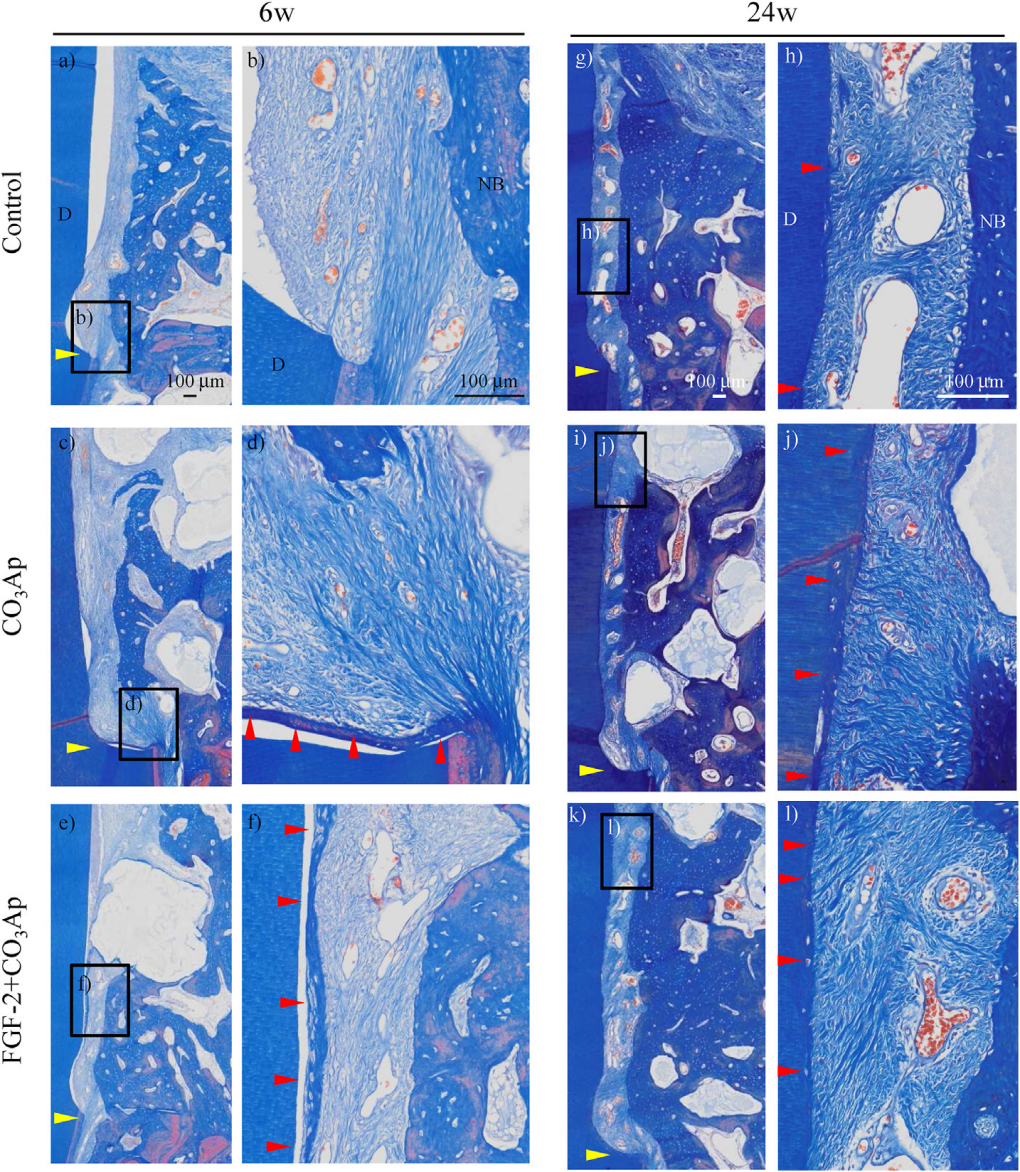
**Histology of the regenerated tissue on the root surface**. Representative photomicrographs of Azan staining from the control (a, b), CO_3_Ap (c, d), and FGF-2+CO_3_Ap (e, f) groups at 6 weeks and the control (g, h), CO_3_Ap (i, j), and FGF-2+CO_3_Ap (k, l) groups at 24 weeks. D: dentin, NB: new bone, yellow arrowheads: defect bottom, red arrowhead: new cementum. Scale bar represents 100 μm.

Enlarged histological images of the root surface are presented in Fig. 7. At 6 weeks, new connective tissues between the dentin and the new bone were immature structure in which the fiber ran vertically, and no new cementum was observed in the control group (Fig. 7b). However, in the CO_3_Ap group, a new cementum and a PDL where fibers were embedded into the new cementum slightly formed on the apical part of the root surface of the defect (Fig. 7d), and an even greater amount of those formation was observed in some defects of the FGF-2+CO_3_Ap group (Fig. 7f). At 24 weeks, a new cementum and a PDL formed in all groups, and their formation in the CO_3_Ap and FGF-2+CO_3_Ap groups extended upwards (Fig. 7h–l). Ankylosis did not occur in any of the three groups.

**Fig. 8:**
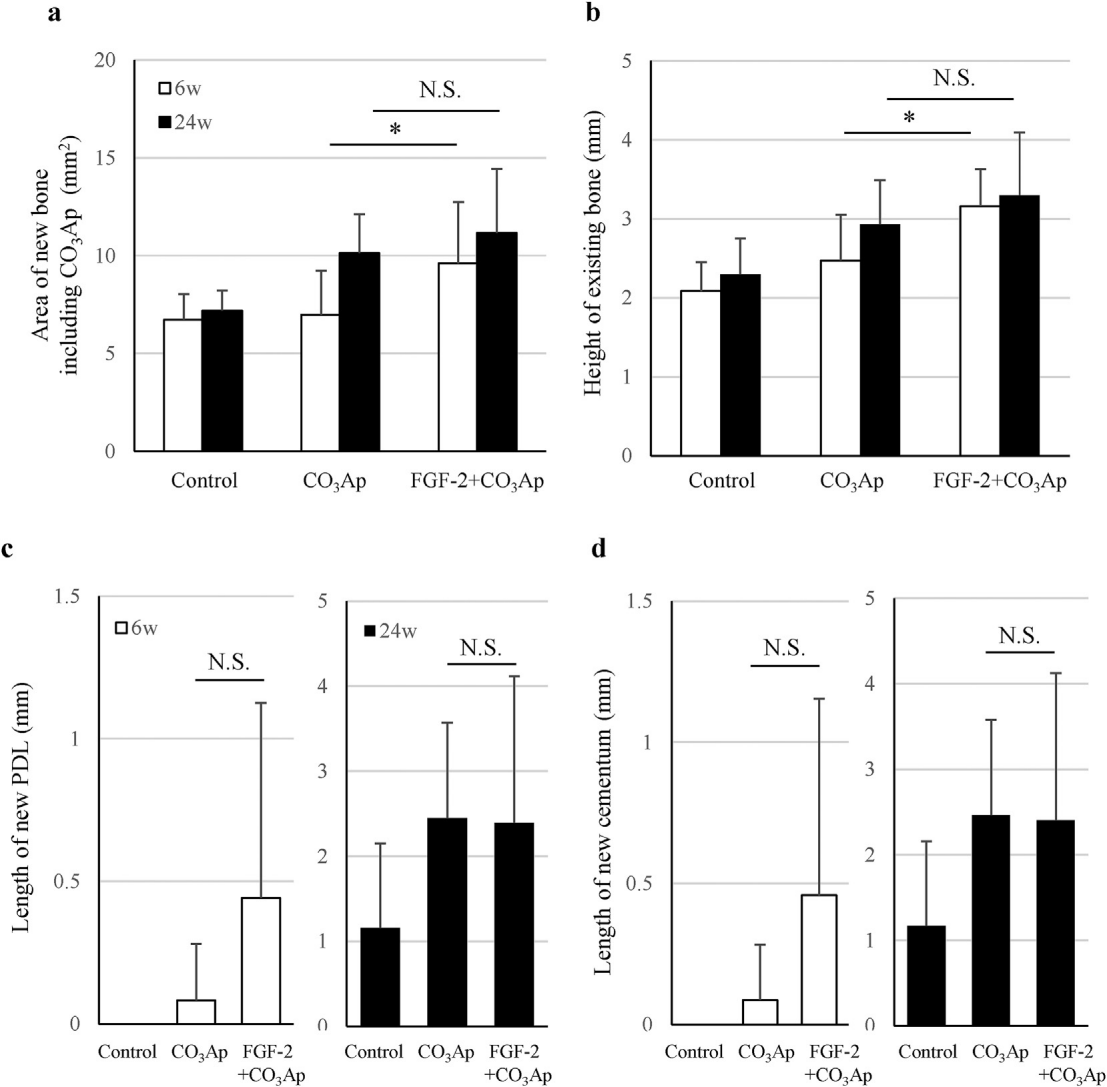
**Histological analysis of the regenerated tissue**. The area of the new bone including CO_3_Ap in the defect site (a), the height of the bone on the mesial side of the defect (b), and the length of the new PDL (c) and cementum (d) were measured. All results are expressed as mean +SD. Control, n = 4; CO_3_Ap, n = 8; FGF-2+CO_3_Ap, n = 8 at each time (white column: 6 weeks, black column: 24 weeks). ∗: *P* < 0.05, NS: not significant (CO_3_Ap vs. FGF-2+CO_3_Ap, a, and b, paired *t*-test at each time; c, and d; Wilcoxon's rank sum test at each time).

Quantitative analysis revealed that the area of the new bone including CO_3_Ap in the defect site and the height of the existing bone on the mesial side of the defect site were great in the order of the FGF-2+CO_3_Ap, CO_3_Ap, and control groups (Fig. 8a and b). With respect to the aforementioned two parameters, a significant difference between the CO_3_Ap and FGF-2+CO_3_Ap groups at 6 weeks was observed. The length of the new PDL and cementum in the FGF-2+CO_3_Ap group was greater than that in the CO_3_Ap group at 6 weeks (Fig. 8c and d). At 24 weeks, the length of the new PDL and cementum was similar between the CO_3_Ap and FGF-2+CO_3_Ap groups.

## 4 Discussion

In this study, we created a beagle dog model of one-wall periodontal defect as a post-surgery model of severe periodontitis. In this model, the combined use of FGF-2 and CO_3_Ap, compared with CO_3_Ap alone, induced regenerations with higher values of mineral content (Fig. 2c), volume of the new bone (Fig. 4b), and area of the new bone including CO_3_Ap (Fig. 8a). In addition, the volume of the new bone in each group did not change at 6–24 weeks (Fig. 4b). These findings indicated that the bone formation was enhanced by the combined use of the FGF-2 and CO_3_Ap and that the regeneration quantity would already be defined by the reactions before 6 weeks in this model. Besides, the μCT analysis at 6 weeks revealed that the CO_3_Ap volume of the FGF-2+CO_3_Ap group was significantly lower than that of the CO_3_Ap group (Fig. 4c). Therefore, it was suggested that the replacement of CO_3_Ap granules with new bone was accelerated by FGF-2 from just after surgery to 6 weeks.

From another point of view, the μCT analysis revealed that the new bone density was similar in all groups at 24 weeks, although the new bone density of the FGF-2+CO_3_Ap group was slightly lower than that of the CO_3_Ap group at 6 weeks (Fig. 4d). It was considered that the new bone induced by the combined use of theFGF-2 and CO_3_Ap followed a maturation process equivalent to that formed by spontaneous healing (control group), although a temporary delay in maturation associated with the promotion of formation was observed. Taken together, such increased new bone formation and early new bone replacement with following spontaneous healing process are presumed to make regenerated tissue closer to normal tissue, and to be advantageous for long-term tissue retention.

These effects of the combined use of the FGF-2 and CO_3_Ap were considered to be caused by the stimulatory effects of FGF-2 accompanied by the high osteoconductivity of CO_3_Ap. FGF-2 promotes angiogenesis [2], migration [3,4], and proliferation [5] of PDL cells and enhances the proliferation of osteogenic progenitor cells from the bone marrow [[6], [7], [8]]. It has been reported that FGF-2 absorbed by CO_3_Ap was slowly released [25]. Therefore, we speculated that the gradual release of FGF-2 adsorbed on CO_3_Ap further enhances three-dimensional migration and proliferation and that the formation of regenerated tissue in the defect site is promoted not only to fill the gap between the CO_3_Ap granules but also to push them out. This speculation was supported by the data in the radiographic analysis that the gap without granules was observed along the existing bone (Fig. 1 red arrowheads) and that the mineral density of the FGF-2+CO_3_Ap group decreased at 0–3 weeks while the mineral area increased (Fig. 2a and b).

Contrary to the above distinct effects, for a new attachment tissue consisting of a new PDL and a new cementum, the formation in the FGF-2+CO_3_Ap group was greater than that in the CO_3_Ap group at 6 weeks (Fig. 8c and d). This data suggested the possibility that the formation of a new attachment tissue is accelerated by FGF-2. This facilitation plays a significant role in eliminating invasion of the gingival tissue and promoting formation of a new attachment tissue. We have previously reported that FGF-2 alone significantly promoted the formation of PDL and cementum in a beagle dog model of two- and three-wall periodontal defect [12,13]. However, in this study, no significant difference between the CO_3_Ap and FGF-2+CO_3_Ap groups was observed probably due to the variability of the study and/or the space-making ability of CO_3_Ap itself, as indicated by the fact that the length of a new PDL and cementum was similar in both groups at 24 weeks.

Besides, in terms of the quantity of the PDL, we observed that the new PDL in the three groups, including the control group, was periostin-positive (Supplemental Fig. S3). Periostin, which is strongly expressed in mature PDL, plays a significant role in the remodeling of collagen fibers in the mechanically loaded region, and its expression increases with mechanical loading in new tissues; thus, it is a marker for PDL regeneration [26,27]. Accordingly, the new PDL induced by FGF-2 and CO_3_Ap may follow a maturation process similar to spontaneous healing.

Finally, the effect of the combined use of the FGF-2 and CO_3_Ap on the existing bone was examined. The height of the existing bone in the FGF-2+CO_3_Ap group was greater than that in the CO_3_Ap and control groups after 6 weeks (Fig. 2d). This may be because the resorption of the existing bone adjacent to the defect was suppressed due to the formation of the new bone in the defect site. Furthermore, it was thought that FGF-2 may have acted on the existing bone. Because FGF-2 has been reported to stimulate not only the formation of a new bone but also the bone metabolism of the existing bone *via* intravenous or intraosseous administration [9], the metabolism balance of the existing bone adjacent to the defect may have been maintained with the predominant state of bone formation. This activity is supported by the fact that the BV and TV in the FGF-2+CO_3_Ap group were significantly higher than those in the CO_3_Ap group (Fig. 5a and b). Furthermore, the difference of the BV and TV between the CO_3_Ap and FGF-2+CO_3_Ap groups may have not been due to the size of the bone marrow cavity but rather the suppression of resorption of the outer bone in contact with the gingival tissue as there was no difference in BV/TV at 24 weeks among all groups (Fig. 5c).

This study demonstrated that the combination of FGF-2 and CO_3_Ap was effective not only in enhancing new bone formation and replacing scaffold but also in maintaining the existing bone adjacent to the defect site in a beagle dog model of one-wall periodontal defect as a post-surgery model of severe periodontitis. Additionally, new periodontal tissues induced by FGF-2 and CO_3_Ap may follow a maturation process similar to that formed by spontaneous healing. Based on the above, this combination treatment may be considered to provide functional and ideal regeneration by securing a regeneration space; activating periodontal regeneration consisting of the bone, PDL, and cementum; and even balancing the bone metabolism of the existing bone around the defect site. Furthermore, it is possible that the beneficial effect of the combined use of the FGF-2 and CO_3_Ap is clearer in patients, because periodontal regeneration in humans is said to be slower and to a lesser degree compared with beagle dogs. Therefore, further standardized clinical trials are warranted to elucidate the usefulness of the combination of FGF-2 and artificial scaffold.

## 5 Conclusions

The combination of FGF-2 and CO_3_Ap was effective in enhancing new bone formation, replacing scaffold, and maintaining the existing bone adjacent to the defect site in a beagle dog model of one-wall periodontal defect. Additionally, new periodontal tissues induced by FGF-2 and CO_3_Ap may follow a maturation process similar to that formed by spontaneous healing. This suggests that the combined use of FGF-2 and CO_3_Ap would promote periodontal regeneration in severe bony defects of periodontitis patient.

## Author contributions

SM, TNT, JA, MK, and TH conceived this project and developed the protocol of this study. TNT, JA, and TH conducted the experiments and analyzed the data. TNT was responsible for coordinating the entire experiments and the histological analysis. JA performed surgery and μCT analysis. TH conducted radiographic analysis. TNT, JA, MT, TH, and SM interpreted and analyzed the data. TNT wrote the manuscripts with the help and comments from all authors.

## Declaration of competing interest

SM received research grants from Kaken Pharmaceutical Co., Ltd., and SM and MK accepted a position as a medical adviser. TNT, JA, and TH are employees and stockholders of Kaken Pharmaceutical Co., Ltd.

## Footnotes

Appendix A Supplementary data to this article can be found online at https://doi.org/10.1016/j.reth.2023.04.002.
