# Supplementary material for: Effects of combined application of fibroblast growth factor (FGF)-2 and carbonate apatite for tissue regeneration in a beagle dog model of one-wall periodontal defect
Source: Regen Ther. 2023 Apr 22;23:84–93. doi: 10.1016/j.reth.2023.04.002 (PMC10141504; doi:10.1016/j.reth.2023.04.002)
Supplement: Multimedia component 1 [file mmc1.pptx]

## Slide 1
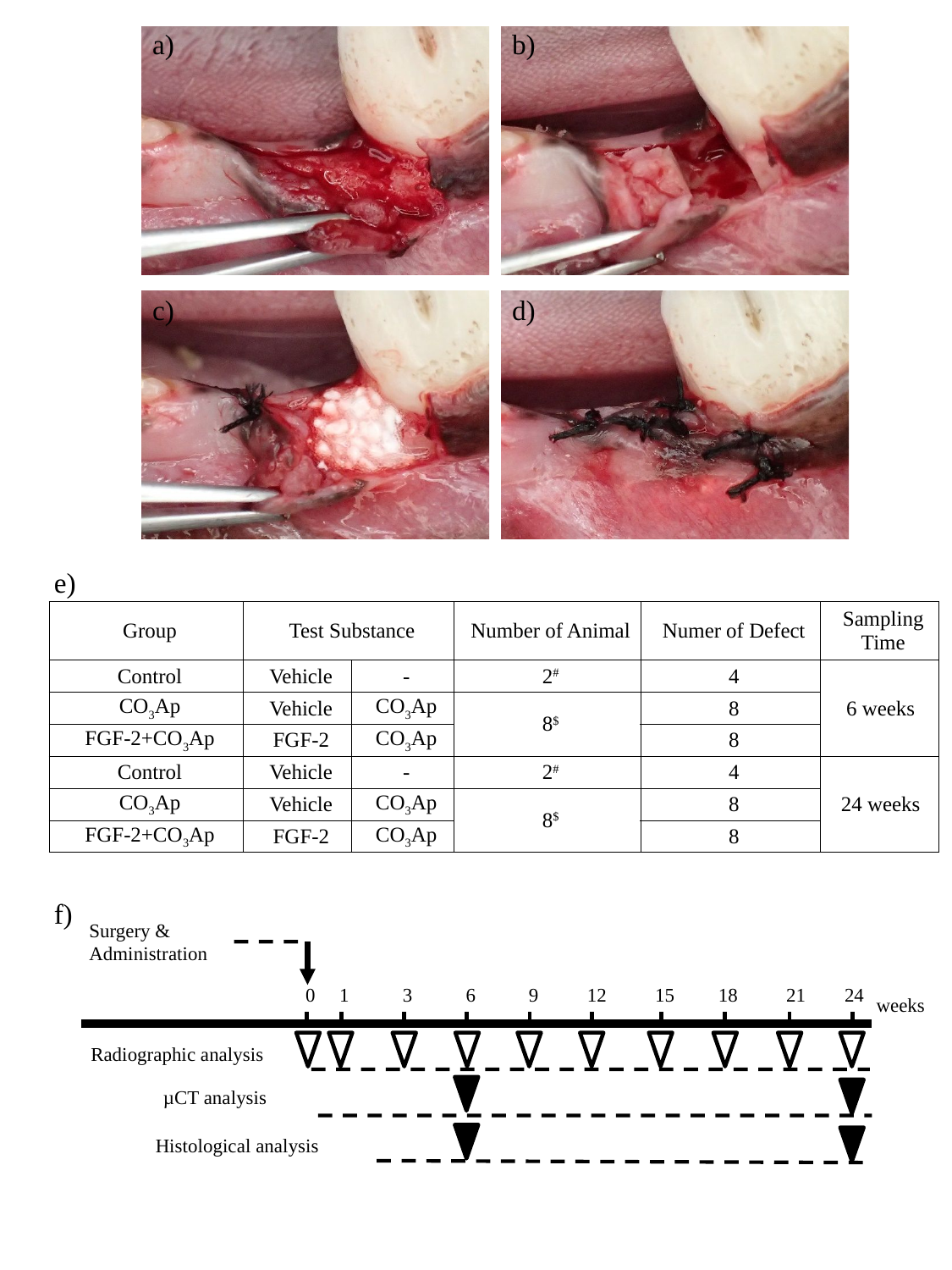

a)
b)
c)
d)
e)
| Group | Test Substance | | Number of Animal | Numer of Defect | Sampling Time |
| --- | --- | --- | --- | --- | --- |
| Control | Vehicle | - | 2# | 4 | 6 weeks |
| CO3Ap | Vehicle | CO3Ap | 8$ | 8 | |
| FGF-2+CO3Ap | FGF-2 | CO3Ap | | 8 | |
| Control | Vehicle | - | 2# | 4 | 24 weeks |
| CO3Ap | Vehicle | CO3Ap | 8$ | 8 | |
| FGF-2+CO3Ap | FGF-2 | CO3Ap | | 8 | |
f)
Surgery &
Administration
0 1 3 6 9 12 15 18 21 24
Radiographic analysis
µCT analysis
Histological analysis
weeks
