# Supplementary figures and images for: Effects of combined application of fibroblast growth factor (FGF)-2 and carbonate apatite for tissue regeneration in a beagle dog model of one-wall periodontal defect
Source: Regen Ther. 2023 Apr 22;23:84–93. doi: 10.1016/j.reth.2023.04.002 (PMC10141504; doi:10.1016/j.reth.2023.04.002)

## Slide 1
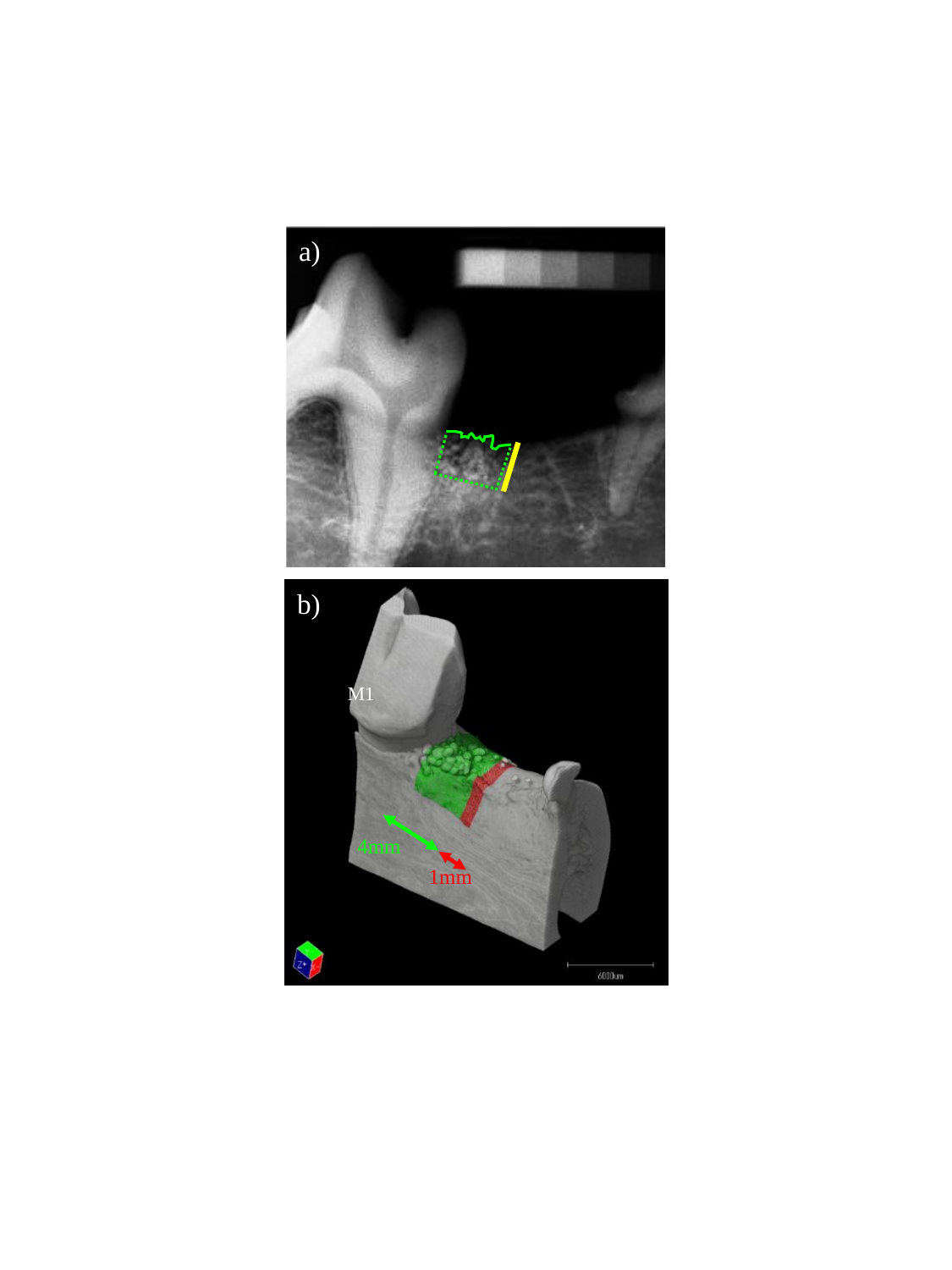

a)
M1
4mm
1mm
b)

Supplement: Multimedia component 2 [file mmc2.pptx]

## Slide 1
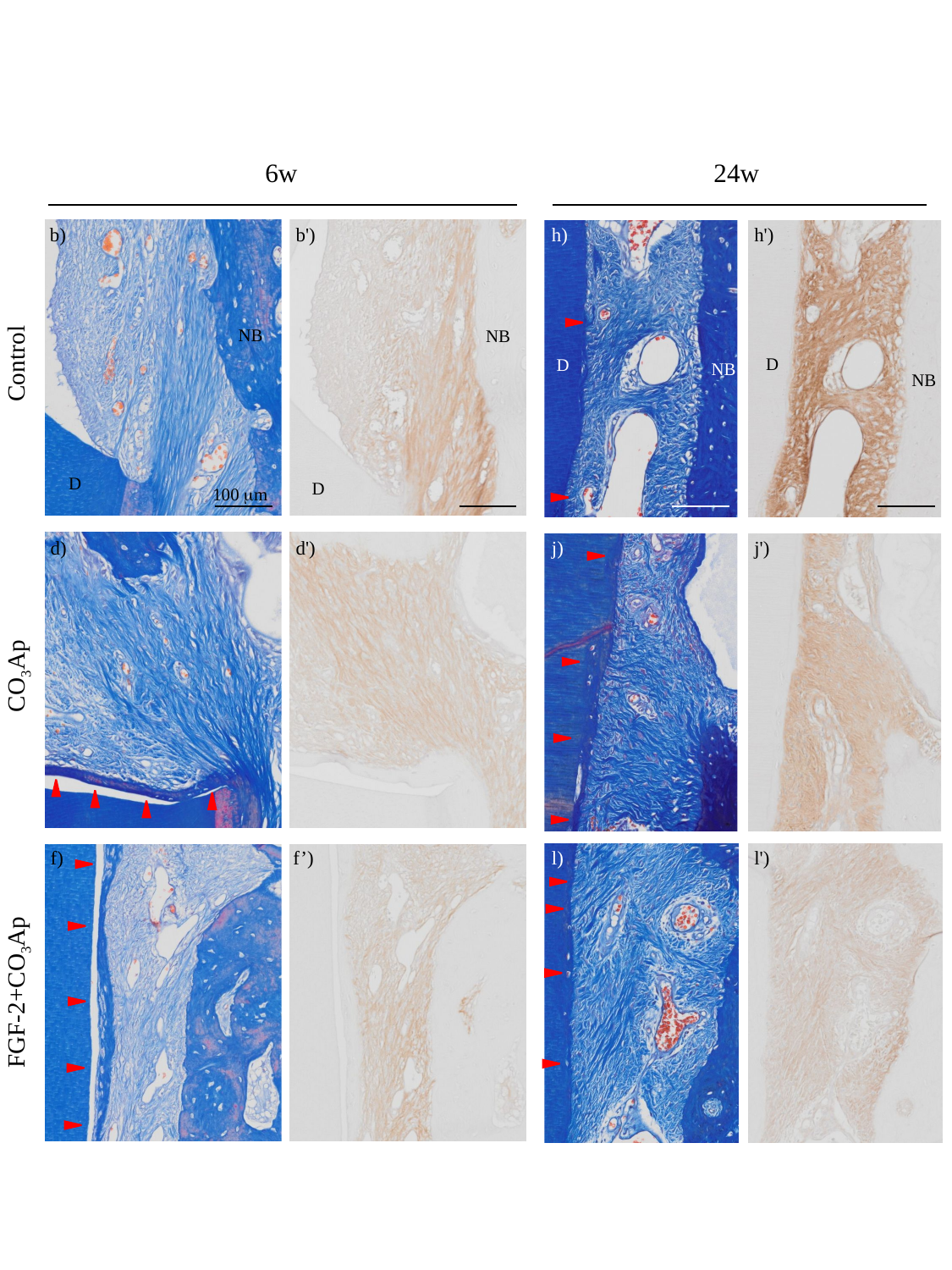

6w
24w
b)
b')
h)
h')
NB
NB
Control
D
D
NB
NB
D
D
100 mm
d)
d')
j)
j')
CO3Ap
f)
f’)
l)
l')
FGF-2+CO3Ap

Supplement: Multimedia component 3 [file mmc3.pptx]
